# Cardioprotective Effects of Nutraceuticals: Focus on Omega-3 Polyunsaturated Fatty Acids

**DOI:** 10.3390/nu13093184

**Published:** 2021-09-13

**Authors:** Grazyna Sypniewska, Stefan Kruszewski

**Affiliations:** 1 Department of Laboratory Medicine, Collegium Medicum, Nicolaus Copernicus University, 85-094 Bydgoszcz, Poland; 2 Department of Biophysics, Collegium Medicum, Nicolaus Copernicus University, 85-094 Bydgoszcz, Poland; skrusz@cm.umk.pl

Cardiovascular diseases are the leading cause of mortality worldwide. Dyslipidemia, in particular elevated circulating low-density lipoprotein cholesterol (LDL-C), has been demonstrated as a causal factor of atherosclerosis. LDL cholesterol-lowering therapies reduce cardiovascular events; however, there is increasing evidence that elevated levels of other lipoprotein particles, containing mainly triglycerides (TG), are associated with the remaining residual cardiovascular risk [1,2,3]. Chylomicrons, very low density lipoproteins (VLDL), intermediate-density lipoproteins (IDL) and their remnants called triglyceride-rich lipoproteins (TRL) may have a direct effect on atherosclerosis as they can enter the arterial wall and cause the development of arterial plaque [4]. All TRL contain cholesterol which is accumulated in the arterial wall and have proinflammatory effects [5]. Different pharmacological therapies can be used to lower triglyceride-rich lipoproteins such as statins, fibrates and PCSK9 (proprotein convertase subtilisin-kexin 9) inhibitors, but their effects are modest, particularly in patients with obesity, type 2 diabetes and metabolic syndrome. On the other hand, high-dose omega-3 polyunsaturated fatty acids (*n*-3 PUFA) and some newer therapeutic agents have been shown to markedly reduce TRL [5]. The cardioprotective effects of different types of diets such as low-fat diets, vegan diets and Mediterranean diets, rich in diverse components of plant origin or rich in seafood, in the primary and secondary prevention of cardiovascular diseases have been recently reviewed.

The PREDIMED (Prevention with Mediterranean diet enriched with nuts or olive oil) trial was performed in a large population of older overweight or obese subjects (48% with type 2 diabetes) at high cardiovascular risk. This study showed that major adverse cardiovascular events (MACE) after almost 5 years of follow-up were not associated with elevated levels of LDL-C but with elevated levels of triglycerides and remnant cholesterol, independent of other risk factors [5]. The risk of MACE at LDL-C >100 mg/dL and remnant-C ≤ 30 mg/dL was much lower than the risk at LDL-C ≤ 100 mg/dL and elevated remnant-C > 30 mg/dL.

The association between higher intake of *n*-3 PUFA and the lower risk of developing cardiovascular diseases was shown through several observational studies; unfortunately, the beneficial cardioprotective effects of *n*-3 PUFA in primary prevention were not enough, sufficiently demonstrated in long-term randomized control trials [6]. In contrast, a number of clinical studies on the effects of *n*-3 PUFA were performed in large populations of subjects at high cardiovascular risk.

Several randomized controlled trials have been undertaken to evaluate the effects of omega-3 polyunsaturated fatty acids on the reduction of hypertriglyceridemia and the atherosclerotic cardiovascular disease (ASCVD) residual risk in patients treated with statins; however, their results seem contradictory. REDUCE-IT (Reduction of Cardiovascular Events with Icosapent Ethyl-Intervention Trial) was the first prospective, placebo-controlled randomized trial including almost 8200 participants at high cardiovascular risk (established cardiovascular disease, type 2 diabetes), well controlled with statins and with moderately elevated triglyceride levels to assess the effects of high-dose omega-3-fatty acids (ethyl eicosapentaenoic acid) on the reduction in the ASCVD residual risk [7,8]. This long-term study demonstrated that icosapent ethyl given at a dose of 4 g per day (2 g twice daily with meals) significantly reduced ASCVD events over and above statin therapy. With reference to the lipid biomarker, administration of high-dose icosapent ethyl markedly reduced TG levels and, to a smaller extent, non-HDL-C and apolipoprotein B levels. Interestingly, the REDUCE-IT trial showed that icosapent ethyl had a beneficial effect, significantly lowering not only TG levels but also the levels of inflammatory marker C-reactive protein (CRP). As endothelial dysfunction and inflammation play a central role in the development and progression of atherosclerosis associated with adverse cardiovascular events, it was suggested that the effect of icosapent ethyl may, in part, result from its pleiotropic action involving inflammation, improvement in endothelial function and coronary plaque stabilization. Whatever the precise mechanisms are by which high-dose icosapent ethyl exerts its effects, they do not undermine a highly significant clinical benefit in reducing the residual cardiovascular risk in hypertriglyceridemic patients presenting with concomitant metabolic disorders. 

The REDUCE-IT study was criticized by several groups of researchers [9,10]. Another similar long-term clinical trial, STRENGTH (Statin Residual Risk in High Cardiovascular Risk Patients with Hypertriglyceridemia), demonstrated contradictory results [10]. This study showed no significant difference in MACE after administration of omega-3-fatty acids in a carboxylic acid formulation [10]. The STRENGTH trial enrolled 12,000 patients at high cardiovascular risk given a mixture of omega-3 FA in a carboxylic acid (CA) formulation (eicosapentanoic acid (EPA) and docosahexaenoic acid (DHA)) at a dose of 4 g daily. Among the statin-treated patients at high cardiovascular risk, the addition of the omega-3 CA supplement, compared with corn oil as placebo, resulted in no significant difference in a composite outcome of major adverse cardiovascular events. According to the authors, these findings do not support the use of omega-3 fatty acids in a carboxylic formulation to reduce major adverse cardiovascular events in high-risk patients. 

Another recent study (OMEMI) on the effects of 2 years of *n*-3 fatty acid supplementation was performed in a group of elderly patients who recently survived acute myocardial infarction [11]. This randomized multicenter clinical trial did not show a reduction in cardiovascular events in patients supplemented with 1.8 g/daily of an *n*-3 polyunsaturated fatty acid (PUFA) mixture of EPA and DHA added to standard medical care when compared to patients receiving corn oil as placebo. In this study, neither a reduction in the primary endpoint (nonfatal AMI, revascularization, stroke, all-cause death or hospitalization due to heart failure) nor a decrease in the secondary outcome (new atrial fibrillation) occurred in patients supplemented with *n*-3 PUFA compared to these in the placebo. It is worth noting that the population studied in the OMEMI clinical trial involved a rather small group of elderly post-myocardial infarction patients with almost normal triglyceride levels, and the duration of observation was relatively short. 

The conflicting results reported thus far on the effects of *n*-3 polyunsaturated FA, in a way, may be due to an inability to compare the study protocols and prompted several investigators to raise an important question: “Which of the three matters most: the type of supplement, the dose or the placebo used as a comparator?” [12]. In a comprehensive review published recently, these issues were addressed [13]. The final conclusion of this review definitely supports the use of high-dose omega-3 (EPA + DHA) as a safe therapy to reduce cardiovascular events in different populations at cardiovascular risk [13].

Nonpharmacological therapy with the supplementation of *n*-3 PUFA can also be effective in heart failure prevention and treatment. Several nutraceuticals have shown some effects when used alone or in combination with pharmacological therapy [14]. According to the recommendations of the International Lipid Expert Panel (ILEP), published in 2020, the intake of some nutraceuticals of plant and animal origin, including *n*-3 PUFA, might be associated with improvements in functional parameters in patients with heart failure, particularly subjects in earlier stages of the disease [14]. Finally, the ILEP experts consistently support the usefulness of supplementation with nutraceuticals, combined with pharmacological therapy, to improve management of patients with heart failure [14]. Interestingly, the recent Australian Heart Failure Guidelines stated a positive recommendation solely for *n*-3 PUFA supplementation as the one that has been adequately studied and clearly shown to be effective in patients with heart failure [15]. 

The pleiotropic mechanism of action of polyunsaturated long-chain omega-3-fatty acids (*n*-3 PUFA) involves, among others, an influence on lipid metabolism, and anti-inflammatory and antithrombogenic effects. A very recent study is worth mentioning due to the applied very specific experimental approach. This small study aimed to explore the effects of polyunsaturated long-chain omega-3-fatty acid (EPA and DHA) supplementation on coronary atherosclerosis with the use of imaging technology [16]. In 106 patients at low to intermediate risk, coronary computed tomography angiography was performed to assess the influence of supplementation with PUFA at an average dose of 1 g/day over a period of 2 months to 5 years [16]. The study demonstrated that supplementation with omega-3 PUFA has anti-atherogenic effects in terms of less high-risk, lipid-rich plaques but higher dense fibro-calcific components, which leads to more stabilized plaques. Importantly, these data confirm earlier observations that *n*-3 PUFA exert their effect at the early stage of plaque formation and, at the late stage, reduce plaque instability [9,17].

Recommendations for the dietary content of PUFA in the prevention of cardiovascular diseases relate to both the *n*-3 and *n*-6 classes of compounds. Alpha-linolenic acid is the precursor of EPA and DHA (*n*-3 PUFA), while linoleic acid generates two metabolites: gamma-linolenic acid and arachidonic acid (*n*-6 PUFA). The association of *n*-6 PUFA levels with cardiovascular diseases (CVD) is still a matter of controversy. A large pooled analysis of 30 prospective cohort studies on the association of dietary *n*-6 PUFA levels and cardiovascular events and cardiovascular mortality included 70,000 subjects and 10,000 CVD events [18]. In this study, the circulating and adipose tissue levels of linoleic and arachidonic acids were evaluated in relation to incident coronary heart disease, ischemic stroke and CVD mortality after adjustment for age, sex, race, presence of diabetes, statins and aspirin use, *n*-3 PUFA levels and the presence of a specific polymorphism in the enzyme taking part in the metabolism of linoleic acid, the last item being associated with ischemic stroke. This comprehensive analysis revealed, most of all, that higher serum and tissue levels of linoleic acid were associated with lower CVD risk. Furthermore, the authors suggested that arachidonic acid levels were not related to major cardiovascular events [18]. 

In this Special Issue of *Nutrients,* a study by Park et al. presents the results of an investigation of the causal effects of serum levels of omega-3 or omega-6 polyunsaturated fatty acids on coronary artery disease (CAD) or myocardial infarction risk. The analysis was performed through Mendelian randomization which uses a genetic instrument developed from genome-wide association studies for various serum omega-3 and omega-6 PUFA levels. Regarding the conclusions from earlier observational cohort studies, the findings of Park et al. represent an important step towards a better understanding of the role of *n*-3 and *n*-6 PUFA in cardiovascular health. 

Recent guidelines of different scientific societies, the Canadian Cardiovascular Society, the American College of Cardiology and the American Heart Association recommend dietary therapy high in vegetables, nuts, whole grains, fruits, olive oil and fish oil combined with pharmacological therapy to augment the beneficial effects in patients with cardiovascular diseases [19,20]. It should be emphasized that the use of purified omega-3 EPA is preferentially recommended for patients older than 45 years at high risk and with hypertriglyceridemia [19,20].

At the beginning of 2021, an updated analysis of five earlier randomized controlled trials was published in a journal of the European Society of Cardiology [21]. The new meta-analysis included studies on the dose-dependent effects of long-term *n*-3 PUFA containing fish oil supplementation on cardiovascular endpoints, particularly the onset of atrial fibrillation in hypertriglyceridemic subjects at high risk of cardiovascular disease or with established CVD. In the final conclusion of this meta-analysis, the investigators suggested that supplementation with *n*-3 fatty acids in patients with hypertriglyceridemia and increased CVD risk is associated with a greater risk of atrial fibrillation. Therefore, before the recommendation for supplementation with *n*-3 fatty acids is given, the potential risk should be taken into consideration in individuals susceptible to developing a heart rhythm disorder [21]. Clinicians, nutritionists and patients should be aware that the consumption of widely available, over-the-counter supplements with different formulations of *n*-3 PUFA (EPA or EPA +DHA) or fish oil may not be efficiently effective in the reduction in the CVD risk. 

The prevention of atherosclerosis and its consequences (myocardial infarction, ischemic stroke, peripheral artery disease) should start early in life. Providing education for young adults on the benefits of a healthy lifestyle to prevent or delay chronic diseases such as cardiovascular diseases is the most important way to reduce the cardiovascular risk in adulthood and during aging. Several reports have emphasized the role of nutraceuticals as safe and efficient lipid-lowering agents. The newest statement of the European Atherosclerosis Society Task Force supports the use of combination therapy for lipid lowering in patients at high and very high risk [22].

Dietary interventions have been shown to exert cardioprotective effects such as decreasing inflammation and oxidative stress, improving endothelial function and lowering blood lipids. This Special Issue of *Nutrients* aims to present ongoing studies related not necessarily to the use of PUFA but also to other approaches in the search for the best solutions for cardiovascular health.
